# The cost-effectiveness of tailored smoking cessation interventions for people with severe mental illness: a model-based economic evaluation

**DOI:** 10.1016/j.eclinm.2023.101828

**Published:** 2023-02-01

**Authors:** Richard Mattock, Lesley Owen, Matthew Taylor

**Affiliations:** a Academic Unit of Health Economics, Leeds Institute of Health Sciences, University of Leeds, Leeds, UK; b York Health Economics Consortium, University of York, York, UK; c National Institute for Health and Care Excellence, London, UK

**Keywords:** Cost-effectiveness, Tobacco, Cessation, Smoking, Severe mental illness

## Abstract

**Background:**

Tailored smoking cessation interventions, which combine behavioural and pharmaceutical support, are effective in populations with severe mental illness (SMI). We establish the cost-effectiveness of two tailored interventions in the UK: (i) a bespoke smoking cessation intervention (BSCI) versus usual care, and (ii) integrated tobacco cessation and mental health care (IC) versus standard smoking cessation clinic (SCC) referral.

**Methods:**

This economic evaluation was conducted between January 15th 2019 and August 4th 2022. We adapted a Markov model estimating smoking status, healthcare costs and quality-adjusted life years (QALYs) across the lifetime. Intervention effectiveness and costs were obtained from a systematic review and a meta-analysis. We obtained specific parameter values for populations with SMI for mortality, risk of smoking related comorbidities, and health utility. Uncertainty was analysed in deterministic and probabilistic sensitivity analysis (PSA).

**Findings:**

The BSCI was cost-effective versus usual care with an incremental cost-effectiveness ratio (ICER) of £3145 per QALY (incremental costs: £165; incremental QALYs: 0.05). Integrated care was cost-effective versus SCC with an ICER of £6875 per QALY (incremental costs: £292; incremental QALYs: 0.04). The BSCI and IC were cost-effective in 89% and 83% of PSA iterations respectively. The main area of uncertainty related to relapse rates.

**Interpretation:**

Our findings suggested that the tailored interventions were cost-effective and could increase QALYs and decrease expenditure on treating smoking related morbidities if offered to people with SMI.

**Funding:**

York Health Economics Consortium was funded by the 10.13039/100010377National Institute for Health and Care Excellence to produce economic evaluations to inform public health guidelines.

**Research in context**

**Evidence before this study**

To inform the study design we conducted a systematic review of economic analyses of tailored smoking cessation interventions for populations with severe mental illness (SMI). Searches were conducted on 7th August 2020 in 11 online databases including MEDLINE and Embase, and contained key search terms for smoking cessation, interventions to promote smoking cessation, and mental health conditions including psychiatric, mood, anxiety, personality, and neurodevelopmental disorders. Four relevant records were identified. Three records contained within-trial economic analyses for a bespoke smoking cessation intervention (BSCI) versus usual care across two UK studies (a pilot and main randomised controlled trial). As these analyses were restricted to the pilot/RCT time horizon they did not account for the known lifetime benefits associated with stopping smoking. The fourth study was a US based economic evaluation which found that integrated tobacco cessation and mental health care (IC) was cost-effective versus standard smoking cessation clinic (SCC) referral for veterans with post-traumatic stress disorder.

**Added value of this study**

The aim of this study was to conduct a UK based economic evaluation to inform the public health advisory committee who are updating the National Institute for Health and Care Excellence (NICE) guidelines on tobacco for England and Wales. Our study builds on current economic evidence which was not considered sufficient to inform national guidelines due to limitations with the 12-month time horizon (BSCI) and relevance of the US setting (integrated care). Our methodology adapts a decision analytic model of smoking cessation in the general population by including parameters that are relevant for populations with SMI. To our knowledge this is the first smoking cessation model parameterised specifically for populations with SMI.

**Implications of all the available evidence**

We found that both BSCI and IC are cost-effective in a UK setting, with a very low level of associated decision uncertainty. The additional resources and costs required to deliver tailored interventions to populations with SMI are sufficiently offset by increased rates of smoking cessation and subsequent reductions in smoking related comorbidities, improved length and quality of life, and reduced healthcare expenditure.

## Introduction

The detrimental health and economic impact of smoking is well established. Tobacco smoking increases the risk of multiple long term health conditions.^1^ Smoking is the leading cause of preventable illness contributing to roughly 19% of deaths in UK adults.^2^ Treating smoking-related illness is estimated to cost the National Health Service (NHS) £3 billion per year.^2^^,^^3^

Tobacco smoking is a particular concern in populations with severe mental illness (SMI), such as schizophrenia, bipolar disorder, psychosis, and post-traumatic stress disorder (PTSD). The prevalence of smoking in populations with SMI may exceed 40%.^4^ Populations with SMI typically inhale more nicotine per cigarette and consume more cigarettes per day than the general population, leading to higher levels of nicotine dependence and increased difficulty in quitting.^5^^,^^6^

In the UK, smoking cessation interventions are generally available through the NHS via direct prescriptions in primary care and through referral/self-referral to local stop smoking services (LSSS). Pharmacological interventions including nicotine replacement therapy (NRT), bupropion and varenicline, and behavioural interventions are effective and cost-effective in the general population.^7^ Pharmacological interventions may be equally effective in populations with SMI, as indicated in a meta-analysis of randomised controlled trials (RCTs) where bupropion (in eight RCTs) and varenicline (in five RCTs) significantly increased quit rates at six months. The same meta-analysis reported mixed results for behavioural interventions (in five RCTs) likely due to differences in the interventions including mode of delivery, and the intensity and frequency of sessions.^8^

Whilst standard cessation interventions may demonstrate some efficacy in RCTs, improving treatment for populations with SMI remains a key policy area that could contribute to reducing health inequalities.^7^ A UK cohort study identified mortality risk two to three times higher for SMI versus general populations, with smoking accounting for 70% of the excess mortality.^9^ Additionally, standard cessation interventions for populations with SMI are provided less frequently per GP consultation, and have lower uptake rates when treatment is offered.^10^^,^^11^

Enhanced smoking cessation interventions that are tailored to the needs of individuals have been developed to address the unmet need in populations with SMI.^12^ The smoking cessation for people with severe mental illness (SCIMITAR+) randomised controlled trial (RCT) evaluated a bespoke smoking cessation intervention (BSCI) which increased quit rates compared with usual care.^12^ Meanwhile, integrated care (IC) for US veterans with military-related PTSD reduced smoking at 12 months when compared with standard referral to a stop smoking clinic (SCC).^13^ Both interventions were tailored to the needs of the underlying populations with SMI through: behavioural support sessions delivered by mental health practitioners and modified based on participants' mental health symptoms; and pharmacological smoking cessation support prescribed by the clinicians who deliver participants’ regular mental health care.

Tailored smoking cessation interventions involve consumption of additional healthcare resources when compared to standard interventions. An important question is whether these additional costs represent value for money. Currently, there is insufficient evidence to determine whether tailored smoking cessation interventions for populations with SMI are cost-effective in UK populations. There are two within trial cost-effectiveness analyses for the BSCI but these are restricted to the RCT time horizon, and thus do not account for the known lifetime benefits associated with stopping smoking.^14^^,^^15^ Meanwhile, there is only a US-based cost-effectiveness study of IC which may not be generalisable to the UK health sector.^16^

The aim of this study was to assess the cost-effectiveness of tailored smoking cessation interventions, across the lifetime, for populations with SMI in the UK. The study was conducted to directly inform the National Institute for Health and Care Excellence (NICE) public health advisory committee (PHAC) responsible for updating guidelines on tobacco cessation and harm reduction.^7^

## Methods

### Study overview and ethics

This study was a model-based economic evaluation (cost-utility analysis) with research conducted between January 15th 2019 and August 4th 2022 in the UK. Ethical approval or informed consent from participants was not required since the research does not include human participants. We obtained all information and evidence for the economic model from published literature.

### Economic evaluation methodology and outcome measures

We adopted a UK healthcare perspective and followed methods specified for cost-utility analyses in the NICE methods manual.^17^ Consequently, the outcome measures included: health and social care costs incurred by the National Health Service (NHS) and the Personal Social Services (PSS) reported as £GBP for 2018/19; and quality-adjusted life years (QALYs) which are a generic measure of health combining morbidity (quality of life) and mortality (length of life). Both costs and QALYs were discounted at 3.5% annually.

The key decision outcome was the incremental cost-effectiveness ratio (ICER), calculated by dividing the incremental costs by the incremental QALYs. The tailored smoking cessation interventions were considered cost-effective versus the comparator if the ICER was lower than the NICE recommended threshold of £20,000 per QALY.^17^

### Interventions

A concurrent systematic literature review was conducted by NICE to identify effectiveness evidence of smoking cessation interventions that have been tailored for populations with SMI.^18^ The review identified two such interventions across three studies.

Firstly, a bespoke smoking cessation intervention (BSCI) was compared to usual care in populations with SMI in the SCIMTAR pilot study and SCIMITAR+ RCT.^12^^,^^15^ The BSCI was a combination of pharmacological and face-to-face behavioural support. It was tailored to the needs of individuals with SMI, being delivered by mental health professionals trained in smoking cessation in conjunction with patients, their GP, and their mental health specialist. Pharmacological support was prescribed (at various dosages) by GPs and included nicotine replacement therapy, bupropion and varenicline. Mental health assessments were made prior to setting a quit date, identifying the reasons for smoking in the context of participants' mental illness. Behavioural support included up to 12 face-to-face sessions plus follow up visits tailored to participants' needs and incorporated a range of behavioural change techniques, such as problem solving, relapse prevention and coping mechanisms, identification of relapse triggers, counselling, and goal setting. Sessions were conducted in mutually agreeable locations, often in participants’ homes rather than healthcare settings. The comparator, usual care, was standard NHS stop smoking services which could include pharmacological and behavioural support, but this was not tailored to individual need.^19^

The second intervention was integrated tobacco cessation and mental health care (IC) for US military veterans with PTSD.^13^ Integrated care was provided by psychologists and social workers who delivered participants' PTSD therapy. The intervention included up to five weekly, three booster and monthly follow up behaviour support sessions tailored to address PTSD symptoms that were related to smoking relapse. This included education on tobacco use, skills to quit smoking, and methods for relapse prevention. Additionally, pharmacological treatment such as NRT, varenicline and bupropion were prescribed (at various dosages) by medical staff delivering participants’ PTSD care. The comparator was referral to standard stop smoking support provided to participants in primary care (SCC). The SCC referral provided treatment within six weeks of referral and included the same prescribed pharmacological medication, and non-tailored behavioural support sessions.^13^

### Population

The population was current smokers from the UK with SMI who are making a quit attempt. We define SMI as any mental illness that causes significant functional impairment and substantially limits major life activities.^20^ This definition is consistent with the RCT populations for the BSCI which included people with schizophrenia, psychotic illness, and bipolar disorder^19^; and IC where the included population had clinical-administered PTSD scale (CAPS) scores indicative of severe PTSD.^13^ Our population was also limited to people aged 12 to 100 as this represented the youngest and oldest ages for which smoking related prevalence data were available.

### Statistical analysis

Statistical analysis was not required for this research.

### Economic model

**Fig. 1:**
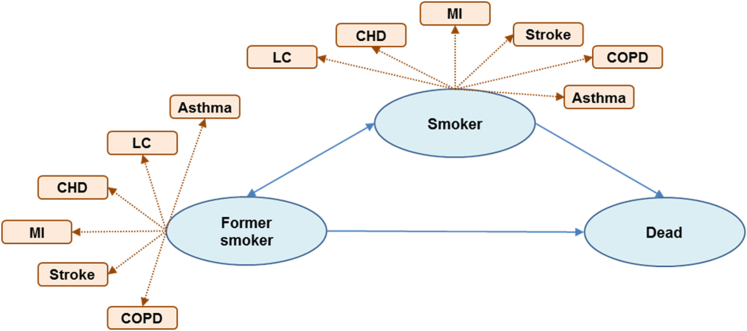
**Model structure.** Legend: For the base model, transitions from “former smoker” to “smoker” did not occur due to application of a combined net rate for cessation and relapse. Bidirectional transitions were included in a sensitivity analysis. CHD = coronary heart disease, COPD = chronic obstructive pulmonary disease, LC = lung cancer, MI = myocardial infarction.

Our analysis adapted a Markov model that was originally developed to inform NICE guidelines on smoking cessation in the general population.^7^ The model includes three health states for “former smoker”, “current smoker” and “dead” (Fig. 1).

The population enter the model in the current smoker health state. The probability of transitioning to the “former smoker” health state at 12 months (i.e. quitting) is determined by effectiveness estimates. After 12 months, populations transition between health states in annual cycles across a lifetime (maximum 100-year) time horizon. Transitions are determined by natural population cessation and relapse rates each year. The probability of death is obtained from age and gender specific all-cause mortality rates, estimated separately for current and former smokers.

Healthcare costs and health-related quality of life reductions (disutility) are applied based on the prevalence of several smoking related comorbidities, estimated separately for former and current smokers and by age and gender. The smoking-related comorbidities are asthma, chronic obstructive pulmonary disease (COPD), coronary heart disease (CHD), lung cancer (LC), myocardial infarction (MI) and stroke.

The model calculates cost-effectiveness using the average lifetime costs and lifetime QALYs across all population age groups. Average population estimates are obtained by calculating costs and QALYs for people at each individual starting age (i.e. aged 12, 13, 14, …, 100) and then combining these estimates across all ages using Office for National Statistics (ONS) population weightings.^21^

### Model parameters

#### Intervention effectiveness

Effectiveness (Table 1) outcomes were biochemically validated abstinence from smoking for 12 months after the initial cessation attempt. For the BSCI analysis, effectiveness was obtained from a meta-analysis in the NICE review which pooled results from the SCIMITAR pilot and SCIMITAR+ RCT.^18^ Effectiveness for IC were obtained directly from the single RCT.^13^

**Table 1:** Intervention effectiveness and costs (£GBP, 2018/19).

	BSCI	Usual care	Source	Integrated care	SCC referral	Source
*Effectiveness: mean (sd)*			[18]			[13]
% abstinence @ 12 months^a^	17.3 (2.4)	11.9 (2.0)		8.9 (1.3)	4.5 (1.0)	
*Intervention costs*			[19]			[16]
Training^b^	£165	£0		–	–	
Supervision^c^	£33	£0		–	–	
Intervention delivery^d^	£243	£0		–	–	
Pharmacotherapies^e^	£94	£30		£362	£251	
Standard care^f^	£53	£67				
Counsellor visits^g^	–	–		£601	£162	
Total costs	£581	£96		£963	£412	

a Biochemically validated quit rates at 12 months post initial cessation attempt pooled across the SCIMITAR+ pilot study and the SCIMTAR+ main study.

b Includes: eight two-day sessions delivered by the National Centre for Smoking Cessation and Training (NCSCT) (total £10,681); time costs for 56 mental health smoking cessation practitioners (MHSCP) (£24,340); printing costs (£109). Total training costs were £43,313 or £165 per BSCI study member.

c 30 min supervision sessions equal to £23, plus CO monitoring costs of £10 per participant.

d Staff time for intervention delivery. Mean sessions = 5.6 per person; mean total delivery time was 492 min per person, total cost = £243 per person.

e All prescribed pharmacotherapies for smoking cessation include NRT (all modes), bupropion and varenicline.

f Consists of cessation consultation with GP/pharmacist, smoking cessation services, and use of NHS helpline.

g Time required for employing a provider of PTSD services.

#### Intervention costs

Intervention costs were obtained from cost-effectiveness studies identified in the NICE review and are reported in Table 1.^18^ Costs were inflated from originally published values to GBP £ 2018/2019 prices using the NHSCII pay and prices indexes.^22^

Costs for the BSCI analysis were sourced from the SCIMITAR+ health technology assessment (HTA).^19^ The BSCI behavioural support costs included time requirements of healthcare professionals for training, supervision, and intervention delivery calculated based on the number of sessions participants attended (mean equal to 5.6 sessions). Costs for the intervention and comparator included standard stop smoking support, such as GP care, counselling sessions, and access to LSSS which were lower for the BSCI than usual care (£53 vs. £67). Meanwhile smoking cessation pharmacotherapies were prescribed more frequently for BSCI than usual care (£94 vs. £30).

Intervention costs in the IC analysis were obtained from a US based cost-effectiveness study.^16^ We converted costs from US$ 2010/11 to £GBP 2010/11 using the average ONS exchange rate for 2010/11 before inflating to £GBP 2018/19.^23^ Resource utilisation for IC included staff time for PTSD therapists who delivered the smoking cessation behavioural support sessions, based on the number of sessions attended by participants, at a mean cost of £601 per participant. The SCC costs included non-tailored counselling sessions, with mean cost of £162 per participant. Mean pharmacotherapy costs were higher for IC (£362) that for SCC (£251).

#### Epidemiological parameters

Where possible, we obtained epidemiological parameter values specific for populations with SMI. We conducted pragmatic literature searches across electronic databases using search terms identified through liaison with information specialists (Appendix 1). Specific values for populations with SMI were identified for mortality risk, risk of smoking related comorbidities, and health-related quality life, Table 2. All other epidemiological parameters remained consistent with the general population model, including for comorbidity costs,^24^, ^25^, ^26^, ^27^, ^28^, ^29^ and utility parameters,^30^, ^31^, ^32^, ^33^, ^34^, ^35^, ^36^ Table 3.

**Table 2:** Epidemiological parameters (SMI specific).

Parameter	Mean	Standard deviation	PSA distribution	Source
Relative risk mortality	2.22	0.02	Lognormal	[37]
Odds ratio smoking related comorbidities	3.10	0.29	Lognormal	[38]
SMI disutility	0.13	0.013	Beta	[32]

**Table 3:** Epidemiological parameters (Not SMI specific).

Parameter	Mean	SD	PSA distribution	Source
Comorbidity costs (per cycle)				
Asthma	£1433	£215	Gamma	[25]
COPD	£636	£95	Gamma	[27]
Coronary heart disease	£1178	£177	Gamma	[29]
Lung cancer	£10,772	£1616	Gamma	[26]
Myocardial infarction	£1135	£170	Gamma	[24]
Stroke	£5618	£842	Gamma	[28]
Utilities				
Base utility for non-smokers	0.88	0.13	Beta	[36]
Current smoker	−0.04	0.01	Beta	[36]
Former smoker	−0.02	0.01	Beta	[36]
Serious mental illness	−0.125	0.013	Beta	[32]
Asthma	0.73	0.11	Beta	[31]
COPD	0.73	0.11	Beta	[33]
Coronary heart disease	0.76	0.03	Beta	[34]
Lung cancer	0.61	0.09	Beta	[30]
Myocardial infarction	0.80	0.12	Beta	[35]
Stroke	0.48	0.07	Beta	[35]
Transition probabilities				
Cessation (current to former smoker)^a^	2.00%	0.3%	Beta	[39]
Relapse (former to current smoker)^b^	0.00%	0%	Beta	[39]

a The cessation rates is a net quit rate which includes people who quit smoking, people who make failed quit attempts, and people who take up smoking (including relapse).

b The relapse rate is set to 0% as relapse is incorporated into the cessation rate.

We did not identify cessation and relapse rates specifically for SMI or mental health populations. Therefore, we applied the base case parameters from the general population model, where the ‘natural’ net rate of cessation is set equal to 2% and incorporates both the expected number of people who quit smoking and relapse from smoking annually.^39^ This meant that after the first 12 months people who remained smokers had a 2% probability of quitting smoking each year, whilst people who had become former smokers had a 0% probability of relapsing. The net rates are not reflective of trajectories of individuals, who are likely to transition between the former smoker and current smoker health states multiple times throughout the model time horizon. Rather, the purpose of the model is to estimate the total number of smokers and former smokers and prevalence of comorbidities in the population over time, which can be approximated using the net cessation and relapse rate. That is, the model does not account for individual pathways, but takes an average cohort approach.

Mortality risk determines the probability of populations transitioning from the former or current smoker health state to the dead health state each cycle. We calculated population-specific mortality risk for current and former smokers with SMI using results from a meta-analysis.^37^ The meta-analysis identified a relative mortality risk of 2.22 for populations with any type of mental health conditions (predominantly anxiety and depression) vs. the general population. We used relative risk estimates obtained from populations with any type of mental health condition as we did not identify any studies that were specific for SMI. The relative risk was multiplied by existing mortality rates for current, former and non-smokers in the general population by age group and gender.^40^

We calculated population-specific risk of smoking related comorbidities for people with SMI using results from a meta-analysis by Daré and colleagues.^38^ This reported the odds of chronic physical disease (diabetes, obesity, cancer, COPD, and CHD) in mental health populations (anxiety, depression, schizophrenia, and bipolar disorder) vs. general populations. The odds ratio, equal to 3.1, was converted to a relative risk for each morbidity using the formula RR = OR/(1 − p + (p ∗ OR)), where p is the underlying absolute probability of each comorbidity in the general population. Each RR was then multiplied by the prevalence of each comorbidity for current and former smokers in the general population model to establish overall occurrence in populations with SMI. General population prevalence estimates for CHD,^41^ COPD,^42^ LC,^43^ MI,^44^ and stroke^45^ and derived estimates for populations with SMI are reported in Appendix 2. Asthma exacerbations were equivalent to general population rates^46^ (i.e. not SMI specific) as the condition description was not consistent with the definition of chronic physical disease in the meta-analysis by Daré and colleagues.^38^

Health-related quality of life for people with SMI was identified from an observational study which used a regression model to estimated mean reduction in SF-6D scores over 12 months versus the general population.^32^ Reductions in HRQoL (i.e. disutility) were equal to −0.196 for mood disorders, −0.043 for anxiety disorders, and −0.278 for substance misuse disorders.^32^ We calculated disutility as the weighted average across all mental health condition specific disutilities using the reported population weights (mood disorder = 38.8%, anxiety disorder = 51.6%, substance misuse disorder = 9.6%).^32^ We applied the resultant weighted disutility of −0.125 equivalently to current and former smoker health states, and by age and gender.

### Deterministic scenario and sensitivity analyses

Three scenario analyses were conducted to investigate key uncertainties in the model parameter values. The first scenario analysis explored the assumptions around intervention effectiveness. Outcomes were expanded to include both biochemically validated and self-reported quitting to 12 months. The effectiveness parameters for this scenario analysis are reported in Appendix 3.

The second scenario analysis added healthcare service utilisation costs to the intervention costs, reported in Appendix 4. Healthcare service utilisation costs were reported in both the existing BSCI and IC cost-effectiveness analyses and included all primary, secondary and community care during the 12-month intervention period.^16^^,^^19^ Following the guidance of experts on the NICE PHAC committee, we excluded service utilisation costs from the base case, only retaining costs related to intervention delivery.^7^ Most service utilisation costs were not expected to directly relate to smoking cessation. The inclusion of unrelated costs can lead to imprecise mean estimates and high levels of variability during sampling (i.e. sampling noise) which may unnecessarily increase decision uncertainty.^47^

Our final scenario analysis altered transition probabilities for relapse and cessation after 12 months and throughout the remaining lifetime. To interrogate the base case assumptions on the use of net transition probabilities, our scenario simultaneously applied: (i) differential rates of smoking cessation and relapse, which may better reflect individual smoking trajectories; and (ii) a substantially higher rate of relapse relative to smoking cessation which may be applicable to populations with SMI. We followed assumptions in an economic model of standard cessation interventions in mental health populations by Wu and colleagues,^48^ who used a relapse rate of 10% based on a UK panel study,^49^ and a cessation rate of 4.1% from a US longitudinal study.^50^

We also conducted extensive univariate deterministic sensitivity analysis by re-estimating cost-effectiveness results after altering parameters from their mean to the value of the upper and lower 95% confidence interval.

### Probabilistic sensitivity analysis

We conducted probabilistic sensitivity analysis (PSA) to quantify the level of uncertainty in the cost-effectiveness results due to uncertainty in the model parameters. When conducting PSA, probabilistic distributions are assigned to each of the model's parameters. Each parameter value is converted from a point estimate to a random variable by drawing from within the parameter's probabilistic distribution. All probabilistic distributions followed recommendations by Briggs and colleagues, that is log-normal for relative risks, beta for probabilities and utilities, and gamma for costs.^31^ The cost-effectiveness results are estimated across multiple iterations, with each iteration including a new set of randomly drawn parameter values from the assigned distributions. The PSA was conducted using 3000 iterations (at which point the results from the simulation were stable i.e. the ICERs did not materially change with each additional iteration).

### Role of the funding source

York Health Economics Consortium was funded by the National Institute for Health and Care Excellence to produce economic evaluations to inform development of public health guidelines. This work was commissioned to inform the NICE public health tobacco guideline update. The study sponsors were involved in the study design, collection, analysis, interpretation of data, writing the report and the decision to submit for publication. All authors had full access to the economic model, verified the data informing the model, and accept responsibility for the decision to submit for publication.

## Results

### Bespoke smoking cessation intervention versus usual care

The BSCI resulted in 55 additional quitters per population of 1000 versus usual care at 12 months. Across the lifetime, the BSCI was cost-effective versus usual care with incremental healthcare costs equal to £165, incremental QALYs equal to 0.05, and an ICER equal to £3145 per QALY (Table 4). The total reduction in costs to treat smoking related comorbidities were £320 per person (stroke = £111, LC = £63, MI = £45, CHD = £20, COPD = £81, Asthma = £0 (Appendix 5)), which almost compensated the intervention costs of £484 per person.

**Table 4:** Cost-effectiveness results.

	Costs			QALYs	ICER
	Intervention	Comorbidities^a^	Total		
**Base case**					
BSCI	£581	£19,770	£20,351	11.57	–
UC	£96	£20,089	£20,187	11.52	–
Incremental	£484	−£320	£165	0.05	£3145
**Scenario 1**^b^					
BSCI	£581	£19,723	£20,304	11.58	–
UC	£96	£20,096	£20,192	11.52	–
Incremental	£484	−£373	£112	0.06	£1837
**Scenario 2**^c^					
BSCI	£8484	£19,770	£28,254	11.57	–
UC	£8763	£20,089	£28,853	11.52	–
Incremental	−£279	−£320	−£599	0.05	Dominant
**Scenario 3**^d^					
BSCI	£581	£20,974	£21,554	11.40	–
UC	£96	£21,083	£21,179	11.38	–
Incremental	£484	−£109	£375	0.02	£19,879
**Base case**					
IC	£963	£20,266	£21,229	11.49	–
SCC	£412	£20,526	£20,192	11.45	–
Incremental	£551	−£260	£292	0.04	£6875
**Scenario 1**^b^					
IC	£963	£19,881	£20,844	11.55	–
SCC	£412	£20,377	£20,788	11.47	–
Incremental	£551	−£492	£56	0.08	£691
**Scenario 2**^c^					
IC	£19,054	£20,265	£39,319	11.49	–
SCC	£19,353	£20,525	£39,878	11.45	–
Incremental	−£299	−£261	−£559	0.04	Dominant
**Scenario 3**^d^					
IC	£963	£21,143	£22,106	11.37	–
SCC	£412	£21,231	£21,643	11.36	–
Incremental	£551	−£88	£463	0.02	£30,223

BSCI = bespoke smoking cessation intervention, UC = usual care, IC = integrated care, SCC = smoking cessation clinic, Dominant = incremental costs < £0, incremental QALYs > 0.

a Annual treatment costs for the following comorbidities: Stroke, lung cancer, myocardial infarction, coronary heart disease, chronic obtrusive pulmonary disease, asthma exacerbation.

b Scenario 1 identifies smoking cessation at 12 months using self-report measures and CO validation.

c Scenario 2 includes all healthcare resource utilisation costs for 12 months post intervention. Healthcare utilisation costs are included as intervention costs.

d Scenario 3 applies natural rate of smoking relapse = 10% and smoking cessation = 4.4% to transitions from 24 months.

**Fig. 2:**
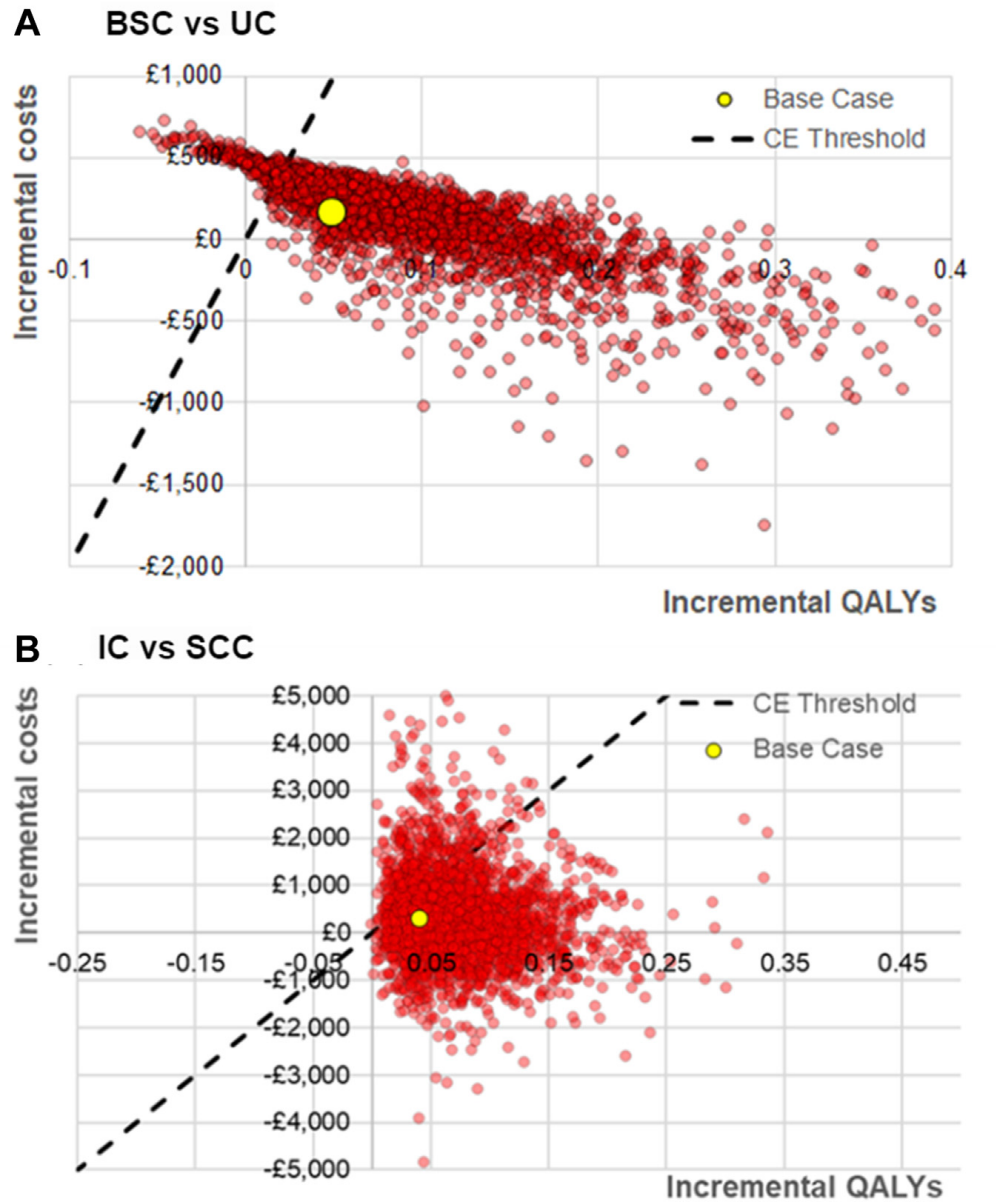
**Probabilistic sensitivity analysis**.

In the PSA, the BSCI was cost-effective in 89% of the 3000 iterations at a cost-effectiveness threshold of £20,000 per QALY (Fig. 2). The probabilistic ICER was £2090 with mean incremental healthcare costs of £173 (SD = £223) and mean incremental QALYs of 0.08 (SD = 0.06). The cost-effectiveness result was robust across all deterministic sensitivity analyses, excluding when restricting the model to a 5-year time horizon (ICER = £54,618 per QALY) and when using the lower 95% confidence interval value for intervention effectiveness at 12 months (ICER = dominated) (Appendix 6).

The BSCI remained cost-effective in the scenario analyses (Table 4) which included: self-reported smoking cessation at 12 months (scenario 1 ICER = £1873 per QALY); and all healthcare service utilisation costs during the first 12 months (scenario 2 ICER = dominant). The BSCI was also cost-effective in the third scenario which increased annual rates of cessation and relapse but resulted in a substantially increased ICER of £19,879 per QALY and higher levels of uncertainty, being cost-effective in 63% of PSA iterations (Appendix 7).

### Integrated care versus smoking cessation clinic

When compared to SCC referral, IC resulted in 44 additional quitters per 1000 at 12 months, incremental lifetime costs of £292 per person, and incremental lifetime QALYs of 0.04 per person. Consequently, IC was cost-effective vs. SCC with an ICER equal to £6875 per QALY (Table 4). Our model estimated that increased rates of smoking cessation following IC would reduce healthcare costs to treat smoking related comorbidities by £260 per person (stoke = £90, LC = £51, MI = £37, CHD = £16, COPD = £66, Asthma = £0 (Appendix 5)).

In the PSA, IC was cost-effective in 83% of the 3000 iterations at a CE threshold of £20,000 per QALY (Fig. 2). The probabilistic ICER was £4218 with mean incremental healthcare costs equal to £292 (SD = £407) and mean incremental QALYs equal to 0.07 (SD = 0.04). Integrated care remained cost-effective in all univariate deterministic sensitivity analyses, excluding when applying effectiveness rates equal to the lower 95% CI (effectiveness IC = 5.3%, SCC = 4.5%, ICER = £58,670 per QALY), and when reducing the time horizon to 5-years (ICER = £81,849), Appendix 6.

Integrated care was cost-effective in two of the three scenario analyses (Table 4). The application of self-reported quit rates resulted in an ICER equal to £1565 per QALY, meanwhile the inclusion of resource utilisation costs during the first 12 months resulted in a dominant ICER but the probability of cost-effectiveness was reduced to 54% in the PSA (Appendix 7). In contrast, IC was not cost-effective in the third scenario analysis which increased relapse and cessation rates (ICER = £30,223, cost-effective in 51% of PSA iterations).

## Discussion

Our analysis indicates both BSCI and IC are cost-effective from a UK healthcare perspective. The tailored interventions are more resource intensive than the comparators requiring substantial time from trained therapists and other healthcare professionals who specialise in smoking cessation. The interventions increased provision of stop smoking support, resulting in increased pharmacological prescriptions and additional behavioural support sessions than were utilised through usual care or standard referral to stop smoking clinics. The additional costs associated with the BSCI and IC were sufficiently offset by increased rates of smoking cessation and subsequent reductions in smoking related comorbidities, improved length and quality of life, and reduced healthcare expenditure.

The interventions were cost-effective in 89% (BSCI) and 83% (IC) of PSA iterations, respectively. Whilst there are no pre-defined PSA thresholds, interventions are typically recommended with increasing frequency as PSA results become more favourable and are almost always recommended if the ICER is below £20,000 per QALY and the PSA results exceed 80%.^51^ Consistent with this, the evidence from our study has been used to inform NICE guidelines, which now recommend offering populations with SMI tailored stop smoking support delivered by mental health specialists.^7^

Whilst both the BSCI and IC were cost-effective, the absolute impact on costs and QALYs was relatively modest, as a large majority of people with SMI continue to smoke after 12 months. Increasing the availability of effective smoking cessation services for populations with SMI remains a key policy and can contribute to reducing health inequalities. Much of the excess morbidity and mortality in populations with mental health conditions is caused by tobacco smoking.^52^ Prevalence rates of smoking are much higher in populations with a mental health diagnosis, despite people having an equal or stronger desire to quit.^52^^,^^53^ People with SMI do not typically engage well with standard smoking cessation services, but do so more readily with tailored interventions.^12^ The analysis shows that tailored interventions would represent a good use of public money and are an effective starting point to address unmet need, helping to prevent people with SMI being left behind.

Our research does not identify why the interventions may be effective and cost-effective. Both were tailored to the needs of the populations with SMI, delivered by mental health specialists, and encouraged additional use of stop smoking services. The interventions differed from the comparators in multiple ways including intensity, timing and frequency of contacts and follow up, the type of behavioural support that was provided, and may have also impacted on participants’ health service utilisation. Tailoring the interventions for populations with SMI is likely to be only one contributing factor leading to increased quit rates. A key area of further research is to identify what works in reducing smoking for populations with SMI and why.

This study was conducted in parallel with a NICE review which identified effectiveness evidence for two tailored smoking cessation interventions (BSCI and IC) and four economic studies related to these interventions.^14^, ^15^, ^16^^,^^19^ Three of the economic studies assessed the cost-effectiveness of BSCI vs usual care informed through the SCIMITAR+ pilot and main RCT.^14^^,^^15^^,^^19^ Whilst BSCI was found to be cost-effective, there was uncertainty in the main RCT analyses with usual care being cost-effective in 24% and 38% of PSA iterations respectively.^14^^,^^19^ Uncertainty was likely due to the time horizon being restricted to the 12-month trial duration, therefore excluding the longer term health benefits associated with smoking cessation. Our study addresses this limitation by modelling the lifetime impact of smoking cessation. We also pool effectiveness estimates from the SCIMTAR+ pilot and main RCT. We find that BSCI is cost-effective with substantially reduced uncertainty, i.e., usual care is cost-effective in only 11% of PSA iterations.

A single economic study was identified for IC which used a lifetime Markov model to establish cost-effectiveness.^16^ The study was not considered directly relevant by the NICE PHAC as the analysis was designed to inform US healthcare providers where healthcare costs associated with smoking were obtained from health care claims and charges databases.^16^ Our study identified the potential impact of IC in terms of costs incurred by the NHS and PSS and is directly relevant to UK decision makers. Our findings that IC is cost-effective versus SCC, is consistent with findings by Barnett and colleagues.^16^

The results from our model are also consistent with cost-effectiveness evidence in the general population.^54^ It is well-established that tobacco smoking is substantially detrimental to health and extremely costly to healthcare systems. Therefore, where interventions are effective in promoting smoking cessation, they are generally found to be cost-effective. Whilst our study was conducted specifically to inform UK policy, the results are likely to be applicable to other settings, given the substantial global burden associated with smoking, and large savings and health benefits that can be achieved by reducing smoking for populations with SMI in general.

We are aware of one other UK-based smoking cessation decision analytic model for UK mental health populations.^48^ When comparing the two models, our model includes additional smoking-related comorbidities and consequently generates additional QALYs and fewer costs, leading to a slight reduction in ICERs for interventions with similar incremental costs and effects.

The main area of uncertainty in the cost-effectiveness results was the transition rates of relapse and cessation after 12 months. We were not able to identify parameter values specific for populations with SMI, therefore, our base case analysis applied transition rates consistent with the general population. Our scenario analysis applied a higher rate of relapse in line with assumptions by Wu and colleagues based on a UK panel study, where the BSCI remained cost-effective, but IC was not cost-effective.^48^^,^^49^

Relapse rates may be higher for SMI versus general populations due to higher levels of nicotine dependence and more challenging social and environmental circumstances^.12^ However, smoking prevalence rates populations with mental health problems, including SMI, are declining.^53^ This suggests the absolute rate of smoking uptake, which includes relapse, does not exceed the absolute rate of smoking cessation, thus supporting assumptions in our base case analysis.

Further, relapse rates typically decline with respect to the time people have remained abstinent.^49^ Our population cohort model doesn't account for individual trajectories. Therefore, the 10% relapse rate in the scenario analysis is applied to all former smokers, irrespective of how long they have remained abstinent. This may result in ICERs for the scenario analysis being overstated, and cost-effectiveness being underestimated. A key area for future research is to quantify long term rates of smoking relapse and cessation in populations with SMI.

There were several other areas of uncertainty in our economic model that may impact the cost-effectiveness results. Tailored interventions for populations with SMI involve increased interaction with mental health services, which may increase overall healthcare utilisation. Following the advice of the NICE PHAC, we did not include all healthcare resource utilisation in the base case analysis due to high levels of sampling noise and imprecision in the RCT.^7^ The cost-effectiveness results did not change when including all healthcare resource utilisation over the first 12 months, however the level of uncertainty increased, for example IC was cost-effective in 83% of PSA iterations in the base case which reduced to 56% in the scenario analysis.

We also note the effectiveness estimates of the BSCI: In the main SCIMITAR+ RCT the effect size was statistically significant at 6 months (p = 0.05), but not at 12 months (p = 0.12)^12^; meanwhile our pooled effectiveness estimate, which includes both the pilot and main SCIMITAR+ sample, is statistically significant at 12 months (p = 0.04).^18^ The inclusion of the pilot study data decreased the uncertainty around the point estimate but does not materially influence the cost-effectiveness results. The incremental difference in intervention effectiveness rates for BSCI and usual care was 5.2% in the main SCIMITAR RCT, and 5.4% using the pooled estimate. Our PSA analysis further accounts for parameter uncertainty by assigning probabilistic distributions using standard errors reported in the SCIMITAR studies. We find that the BSCI is cost-effective versus usual care in 89% of the PSA iterations.

Additionally, our sensitivity analyses include results for populations aged 20 and aged 60 which differ in terms of disease prevalence rates, healthcare costs, and health utility, but not effectiveness estimates. It is possible that intervention effectiveness rates differ across age groups, but this was not reported in either of the RCTs informing the analysis,^12^^,^^13^ and the impact of age on probability of quitting is not consistent in the current literature.^55^^,^^56^ We also note that any differences in effectiveness rates would need to be substantial to alter the cost-effectiveness results.

Finally, there are several other potential benefits associated with smoking cessation that were not included in the analysis. The model includes only six of the many diseases caused by smoking. Improved recovery from other healthcare interventions such as surgery, impact on other people's smoking behaviour and the effects of second-hand smoke are also not included. Additionally, we do not account for any of the health benefits associated with reduced tobacco consumption when this doesn't result in fully quitting. Further we estimated mortality risk using data from populations with general mental illness rather than specifically for SMI. The exclusion of these factors, due to a lack of reliable data and resource limitations, suggests that the current analysis may underestimate the real benefits of smoking cessation. This analysis indicates that both tailored mental health interventions are cost-effective in a UK setting. The inclusion of additional benefits would only act to reduce the estimated ICERs further below the £20,000 per QALY threshold.

## Contributors

RM was involved in all stages of research including planning, developing the economic model, and leading writing of the manuscript. LO contributed to planning the analysis, interpreting results, and writing the manuscript. MT contributed to planning the analysis, developing the economic model, and writing the manuscript. All authors had full access to the economic model, verified the data informing the model, and accept responsibility for the decision to submit for publication.

## Data sharing statement

No primary data was obtained for this analysis. The economic model utilises secondary data with all sources available from published literature.

## Declaration of interests

There are no conflicts of interest.
