# Supplementary material for: The cost-effectiveness of tailored smoking cessation interventions for people with severe mental illness: a model-based economic evaluation
Source: eClinicalMedicine. 2023 Feb 1;57:101828. doi: 10.1016/j.eclinm.2023.101828 (PMC9925867; doi:10.1016/j.eclinm.2023.101828)

Supplementary Material Contents

Appendix 1: Pragmatic Literature Search Strategy 2
Appendix 2: Comorbidity Prevalence Rates 3
Appendix 3: Intervention Effectiveness (biochemically validated or self-reported) 4
Appendix 4: Intervention costs (£GBP, 2018/19), including healthcare service utilisation 4
Appendix 5: Full Cost Breakdown 5
Appendix 6: Univariate Sensitivity Analyses 6
Appendix 7: Scenario Analysis PSA results 7

Appendix 1: Pragmatic Literature Search Strategy

Pragmatic literature searches were conducted in online databases, to update model input parameters for SMI populations. The searches were conducted by information specialists at York Health Economics Consortium (YHEC). The searches combined key terms and synonyms relating to each model parameter, mental health and SMI populations, and where appropriate tobacco smoking or smoking cessation. Searches were conducted in electronic databases including Medline, Embase, Google Scholar, the CEA Registry, and the burden of illness database HEORO. Searches were conducted in an iterative nature, i.e. first conducted on title, then on abstract if no relevant evidence was identified; and similarly using only SMI related search terms, then general mental health related search terms if no evidence was identified.

An example of the search strategy for utility values for SMI populations by smoking status is provided below:

#1	Terms for serious mental illness: ("serious" or "severe" or "chronic" or "persistent") NEAR/2 ("mental health" or "mental-health" or "mental illness" or "mental-illness")
#2	Terms for mental health: "mental health" or "mental-health" or "mental illness" or "mental-illness"
#3	Terms for QALY: "qaly" or "qalys" or ("quality" NEAR/2 "health related" or "health-related")
#4	Terms for disutility: "disutility*" or "dis utility" or "dis utilities" or ("utility" NEAR/5 ("decrement" or "decrease*"))
#5	Terms for smoking status: "smoke*" or "tobacco" or "non-smoke*" or "non smoke*" or "ex-smoke*" or "ex smoke*"
#6	Search 1: Combine terms 1 & (3 or 4) and 5 [title only]
#7	Search 2: Combine terms 1 & (3 or 4) and 5 [abstract]
#8	Search 3: Combine terms 2 & (3 or 4) and 5 [title only]
#9	Search 4: Combine terms 2 & (3 or 4) and 5 [abstract]

Appendix 2: Comorbidity Prevalence Rates

Disease	Age	Prevalence in general population	Relative risk	Prevalence in SMI population	Source
Asthma exacerbation	12 to 15	0.08%	NA	0.08%	[45]
	35 to 64	0.05%	NA	0.05%	[45]
	65+	0.07%	NA	0.07%	[45]
Coronary Heart Disease	12 to 15	0.00%	3.10	0.00%	[40]
	16 to 24	0.10%	3.09	0.31%	[40]
	25 to 34	0.20%	3.09	0.62%	[40]
	35 to 44	0.60%	3.06	1.84%	[40]
	45 to 54	3.60%	2.88	10.38%	[40]
	55 to 64	10.60%	2.54	26.88%	[40]
	65 to 74	20.80%	2.16	44.88%	[40]
	75+	28.60%	1.94	55.40%	[40]
COPD	12 to 15	0.10%	3.09	0.31%	[41]
	16 to 44	1.28%	3.02	3.86%	[41]
	45 to 64	4.15%	2.85	11.83%	[41]
	65 to 74	8.31%	2.64	21.93%	[41]
	75+	8.94%	2.61	23.33%	[41]
Lung Cancer	12 to 44	0.002%	3.10	0.01%	[42]
	45 to 64	0.09%	3.09	0.28%	[42]
	65+	0.75%	3.05	2.28%	[42]
Myocardial Infarction	12 to 34	0.00%	3.10	0.00%	[43]
	35 to 44	0.34%	3.08	1.06%	[43]
	45 to 54	3.15%	2.91	9.16%	[43]
	55 to 64	4.49%	2.83	12.72%	[43]
	65 to 74	11.64%	2.49	29.00%	[43]
	75%	12.90%	2.44	31.47%	[43]
Stroke	12 to 44	0.11%	3.09	0.34%	[44]
	45 to 54	0.89%	3.04	2.71%	[44]
	55 to 64	2.69%	2.93	7.89%	[44]
	65 to 74	6.4%	2.73	17.49%	[44]
	75+	14.89%	2.36	35.16%	[44]

Prevalence rates for SMI populations are obtained by multiplying prevalence rates in general populations by relative risks. Relative risks (RR) are derived using the odds ratio (OR) and underlying prevalence rates (p) as follows: RR = OR/(1-p+(p*OR)). The odds ratio for all smoking related comorbidities was obtained from a meta-analysis by Daré and colleagues and equal to 3.1.[39]

Appendix 3: Intervention Effectiveness (biochemically validated or self-reported)

	**P(abstinence) at 12-months**	**Source**
BSCI	18.19%	[18]
Usual care	11.81%	[18]
Integrated care	15.5%	[13]
SCC referral	7.0%	[13]

Appendix 4: Intervention costs (£GBP, 2018/19), including healthcare service utilisation

	Mean	SE	**Source**
BSCI vs. usual care (incremental) [a]	-£270	£825	[19]
Integrated care [b]	£19,054	£22,265	[16]
SCC referral [b]	£19,353	£22,655	[16]

a: incremental cost for BSCI versus usual including all intervention, prescription, and healthcare service utilization costs. The total incremental costs were adjusted for baseline characteristics using regression analysis, with covariates for age, gender, pre-existing medical conditions, duration since diagnosis of severe mental illness and healthcare costs incurred during 6-months prior to randomization.

b: Aggregated costs including service utilisation. Service utilisation costs include mental health treatment for PTSD and all cause hospital inpatient stay, outpatient visits and pharmacy costs.

Appendix 5: Full Cost Breakdown

	Intervention costs	Comorbidity Costs						Total Costs
		Stroke	LC	MI	CHD	COPD	Asthma	
**Base case**								
BSCI	£581	£9,054	£2,133	£2,249	£3,775	£2,546	£13	£20,351
UC	£96	£9,165	£2,195	£2,294	£3,795	£2,627	£13	£20,187
Incremental	£484	-£111	-£63	-£45	-£20	-£81	-£0	£165
**Scenario 1** [a]								
BSCI	£581	£9,037	£2,123	£2,242	£3,772	£2,535	£14	£20,304
UC	£96	£9,167	£2,196	£2,295	£3,795	£2,629	£14	£20,192
Incremental	£484	-£130	-£73	-£53	-£23	-£94	-£0	£112
**Scenario 2** [b]								
BSCI	£8,484	£9,054	£2,133	£2,249	£3,775	£2,546	£13	£28,254
UC	£8,763	£9,165	£2,195	£2,294	£3,795	£2,627	£13	£28,853
Incremental	-£279	-£111	-£63	-£45	-£20	-£81	-£0	-£599
**Scenario 3** [c]								
BSCI	£581	£9,493	£2,382	£2,419	£3,848	£2,818	£14	£21,554
UC	£96	£9,531	£2,403	£2,435	£3,854	£2,847	£14	£21,179
Incremental	£484	-£37	-£21	-£15	-£7	-£29	-£0	£375
**Base case**								
IC	£963	£9,226	£2,229	£2,319	£3,806	£2,672	£14	£21,229
SCC	£412	£9,317	£2,280	£2,356	£3,822	£2,737	£14	£20,192
Incremental	£551	-£90	-£51	-£37	-£16	-£66	-£0	£292
**Scenario 1** [a]								
IC	£963	£9,092	£2,154	£2,265	£3,782	£2,574	£14	£20,844
SCC	£412	£9,265	£2,251	£2,335	£3,813	£2,699	£14	£20,788
Incremental	£551	-£170	-£97	-£70	-£30	-£125	-£0	£56
**Scenario 2** [b]								
IC	£19,054	£9,226	£2,229	£2,319	£3,806	£2,671	£14	£39,319
SCC	£19,353	£9,316	£2,280	£2,356	£3,822	£2,737	£14	£39,878
Incremental	-£299	-£91	-£51	-£37	-£16	-£66	-£0	-£559
**Scenario 3** [c]								
IC	£963	£9,551	£2,414	£2,443	£3,858	£2,862	£14	£22,106
SCC	£412	£9,582	£2,431	£2,456	£3,864	£2,886	£14	£22,643
Incremental	£551	-£30	-£17	-£12	-£5	-£23	-£0	£463

BSCI = bespoke smoking cessation intervention, UC = usual care, IC = integrated care, SCC = smoking cessation clinic, Dominant = incremental costs < £0, incremental QALYs > 0.

a: Scenario 1 identifies smoking cessation at 12-months using self-report measures and CO validation.

b: Scenario 2 includes all healthcare resource utilisation costs for 12-months post intervention. Healthcare utilisation costs are included as intervention costs.

c: Scenario 3 applies natural rate of smoking relapse = 10% and smoking cessation = 4.4% to transitions from 24-months.

Appendix 6: Univariate Sensitivity Analyses

Deterministic sensitivity analysis: BSCI versus usual care

DSA Scenario	DSA Parameter Value	Absolute (BSCI)		Incremental (BSCI vs. usual care)		
		Costs	QALYs	Costs	QALYs	ICER
Base Case	N/a	£20,351	11.57	£165	0.05	£3,145
Effectiveness	Lower 95% CI RR (0.96)	£20,699	11.52	£512	-0.00	Dominated
	Upper 95% CI RR (2.23)	£19,816	11.66	-£371	0.14	Dominant
Intervention costs	Increase by 25%	£20,496	11.57	£310	0.05	£5,918
	Decrease by 25%	£20,206	11.57	£19	0.05	£372
Time horizon	5 years	£5,407	3.09	£389	0.01	£54,618
Cessation rate	Increase to 5% per year	£18,915	11.80	£260	0.04	£6,939
Relapse rate	Increase to 5% per year	£21,671	11.38	£306	0.03	£10,100
Discount rate	Costs 5%, QALYs 5%	£16,465	9.62	£212	0.04	£5,201
	Costs 1.5%, QALYs 1.5%	£29,231	15.67	£70	0.08	£899
Utility	Same QoL for smokers and non-smokers	£20,351	11.95	£165	0.04	£3,759
Disease costs	Decrease by 25%	£15,409	11.57	£245	0.05	£4,672
	Increase by 25%	£25,294	11.57	£85	0.05	£1,618
Disease disutility	Decrease by 25%	£20,351	11.85	£165	0.05	£3,470
	Increase by 25%	£20,351	9.78	£165	0.09	£1,886
Age of population	Age = 20	£9,967	17.35	£358	0.04	£9,569
	Age = 60	£29,734	7.98	-£22	0.07	Dominant

Deterministic sensitivity analysis: Integrated Care vs SCC

DSA Scenario	DSA Parameter Value	Absolute (IC)		Incremental (IC vs. SCC)		
		Costs	QALYs	Costs	QALYs	ICER
Base Case	N/a	£21,229	11.49	£292	0.04	£6,875
Effectiveness	Lower 95% CI Prob (5.3%)	£21,437	11.46	£499	0.01	£58,670
	Upper 95% CI Prob (14.8%)	£20,886	11.55	-£52	0.10	Dominant
Intervention costs	Increase by 25%	£21,470	11.49	£532	0.04	£12,508
	Decrease by 25%	£20,940	11.49	£2	0.04	£55
Time horizon	5 years	£5,938	3.08	£473	0.01	£81,849
Cessation rate	Increase to 5% per year	£19,645	11.74	£369	0.03	£12,115
Relapse rate	Increase to 5% per year	£22,328	11.33	£406	0.02	£16,444
Discount rate	Costs 5%, QALYs 5%	£17,269	9.56	£330	0.03	£9,947
	Costs 1.5%, QALYs 1.5%	£30,255	15.55	£215	0.06	£3,381
Utility	Same QoL for smokers and non-smokers	£21,229	11.89	£292	0.04	£8,185
Disease costs	Decrease by 25%	£16,162	11.49	£356	0.04	£8,374
	Increase by 25%	£26,295	11.49	£226	0.04	£5,320
Disease disutility	Decrease by 25%	£21,229	11.77	£292	0.04	£7,556
	Increase by 25%	£21,229	9.65	£292	0.07	£4,107
Age of population	Age = 20	£10,544	17.29	£449	0.03	£14,744
	Age = 60	£30,901	7.87	£139	0.06	£2,467

Appendix 7: Scenario Analysis PSA results

	Percentage of iterations strategy is cost-effective			
	Base case	Scenario 1[a]	Scenario 2[b]	Scenario 3[c]
Bespoke smoking cessation intervention	89%	92%	94%	63%
Usual care	11%	8%	6%	37%
Integrated care	83%	94%	54%	51%
Smoking cessation clinic	17%	6%	46%	49%

a: Scenario 1 identifies smoking cessation at 12-months using self-report measures and CO validation.

b: Scenario 2 includes all healthcare resource utilisation costs for 12-months post intervention. Healthcare utilisation costs are included as intervention costs.

c: Scenario 3 applies natural rate of smoking relapse = 10% and smoking cessation = 4.4% to transitions from

Bespoke Smoking Cessation Intervention vs Usual Care

Scenario 1: Self-report and CO validated quit rates

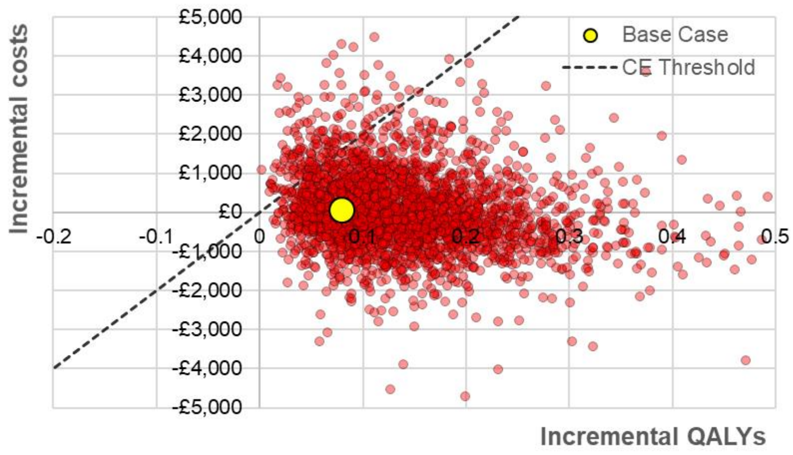

Scenario 2: Includes all healthcare resource utilisation costs during first 12-months

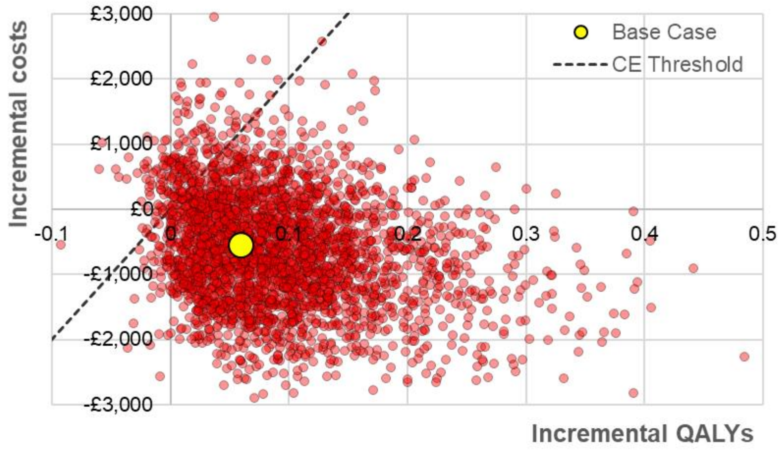

Scenario 3: Adapted relapse and cessation rates

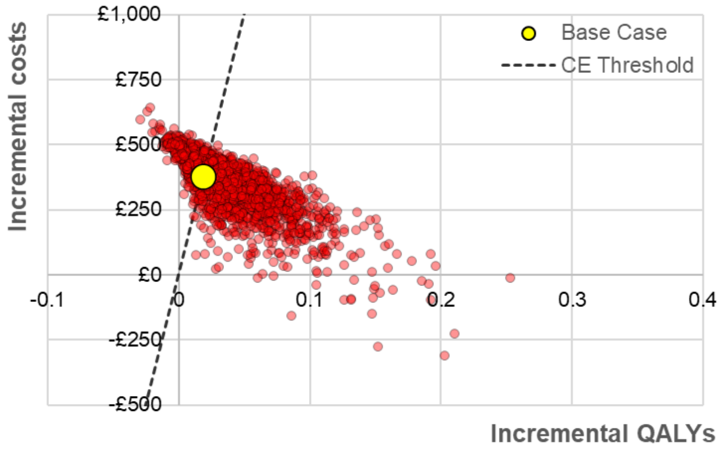

Integrated Care vs Smoking Cessation Clinic

Scenario 1: Self-report and CO validated quit rates

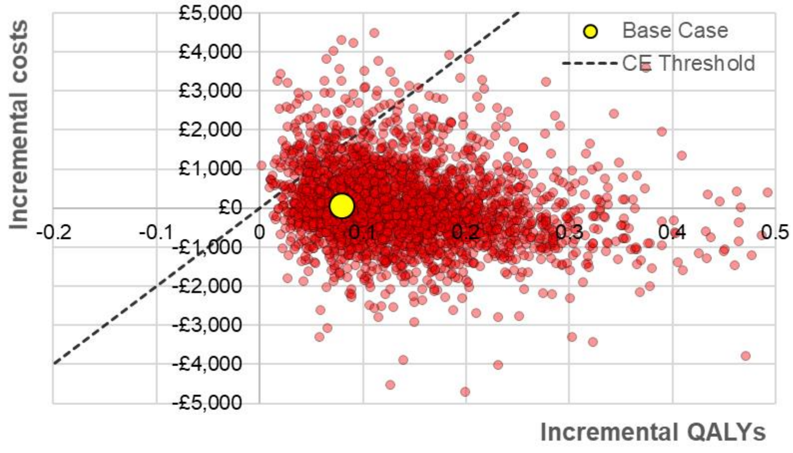

Scenario 2: Includes all healthcare resource utilisation costs during first 12-months

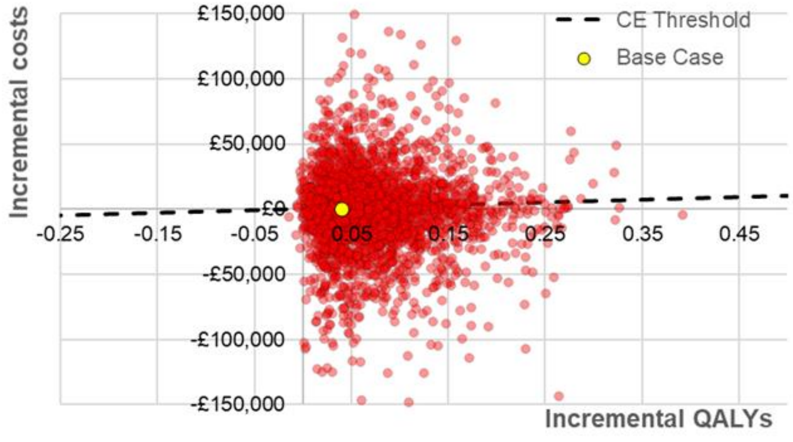

Scenario 3: Adapted relapse and cessation rates

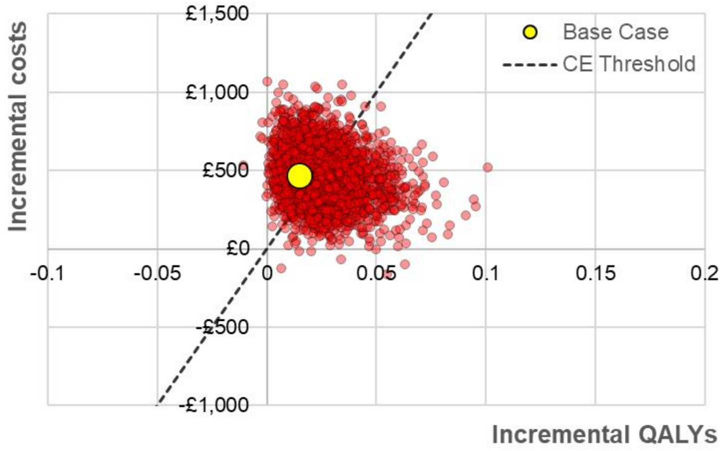

Supplement: Supplementary Material [file mmc1.pdf]
